# Effects of phytoplankton physiology on global ocean biogeochemistry and climate

**DOI:** 10.1126/sciadv.adg1725

**Published:** 2023-07-26

**Authors:** Chia-Te Chien, Markus Pahlow, Markus Schartau, Na Li, Andreas Oschlies

**Affiliations:** ^1^ GEOMAR Helmholtz Centre for Ocean Research Kiel, Düsternbrooker Weg 20, 24105 Kiel, Germany.; ^2^ Kiel University, 24118 Kiel, Germany.

## Abstract

The similarity of the average ratios of nitrogen (N) and phosphorus (P) in marine dissolved inorganic and particulate organic matter, dN:P and pN:P, respectively, indicates tight links between those pools in the world ocean. Here, we analyze this linkage by varying phytoplankton N and P subsistence quotas in an optimality-based ecosystem model coupled to an Earth system model. The analysis of our ensemble of simulations discloses various feedbacks between changes in the N and P quotas, N_2_ fixation, and denitrification that weaken the often-hypothesized tight coupling between dN:P and pN:P. We demonstrate the importance of particulate N:C and P:C ratios for regulating dN:P on the global scale, with marine oxygen level being an important control. Our analysis provides further insight into the potential interdependence of phytoplankton physiology and global climate conditions.

## Teaser

Changes in phytoplankton subsistence quotas induce complex global scale feedbacks between marine nutrient ratios and climate.

## INTRODUCTION

In the 1930s, A. C. Redfield found the similarity between the average ratios of nitrogen (N) and phosphorus (P) in marine dissolved inorganic and particulate organic matter (dN:P and pN:P), which suggests a tight relationship between dissolved and particulate pools (*1*). This similarity is difficult to explain because observations show considerable variations of pN:P and dN:P in time and space. Previous studies have focused mostly on how non–N_2_-fixing phytoplankton N:P (phyN:P) may regulate dN:P under the assumption of a globally constant phyN:P (*2*–*4*). This assumption is at odds with observations revealing that ambient conditions (nutrients, light, and temperature) strongly affect phytoplankton stoichiometry (*5*–*8*). In addition, variations in phyN:P appear to contribute substantially to the pronounced spatiotemporal variability of pN:P across the world ocean (*9*). Thus, a constant phyN:P likely introduces considerable bias to model investigations with regard to the regulation of dN:P.

Maintaining dN:P close to the Redfield ratio could be the result of effective averaging within ecosystems on a seasonal time scale or of large-scale mixing via ocean circulation (*10*). Several physiological and ecological hypotheses also have been proposed to explain the similarity of dN:P and pN:P in view of the variability of phytoplankton C:N:P (*11*, *12*). Nevertheless, a convincing explanation for this phenomenon remains elusive. A useful model approach for elucidating interdependencies between dN:P and pN:P is to combine biogeochemical processes with ocean circulation, i.e., advection and mixing. From a climate perspective, changes in dN:P and pN:P affect marine primary production and the vertical export and remineralization of particulate organic matter (POM) in the ocean, which have an impact on atmospheric carbon dioxide (CO_2_) concentration. Earth system models can be used for testing hypotheses that may explain global-scale phenomena (*13*), such as the relations among dN:P, pN:P, and global climate conditions. Here, we apply the optimality-based plankton ecosystem model (OPEM) that relates the optimal growth of the plankton to ambient environmental conditions while resolving variations in elemental ratios (N:C, P:C, and N:P) of the POM and dissolved inorganic nutrients (*14*, *15*). The OPEM ecosystem contains two functional types of primary producers, (non–N_2_ fixing) phytoplankton and facultative diazotrophs. OPEM allocates cellular resources of primary producers for the acquisition of C, N, and P to maximize the net balanced growth rate. It is implemented in the UVic Earth system climate model (UVic-ESCM), an Earth system model of intermediate complexity that is computationally feasible for a large number of ensemble simulations (*16*). UVic-ESCM contains an atmospheric component and therefore allows examining mutual dependencies between marine biogeochemistry and the global climate.

The effects of variable phytoplankton C:N:P on the global biogeochemistry have received considerable attention in recent years. Variable C:N:P affects surface nutrient distributions and inventories (*10*, *17*–*19*) and could also mitigate the changes in C export under different climate conditions (*20*–*23*). Previous studies have considered how variations in elemental stoichiometry may be linked to the diversity of phytoplankton functional types (*10*, *24*) and how allowing for variable stoichiometry affects future climate projections (*25*).

Here, we revisit the more fundamental question of what drives the similarity between pN:P and dN:P on the global scale. We start out with calibrating a reference simulation for OPEM coupled to UVic-ESCM (UVic-OPEM) against data from the World Ocean Atlas 2013 (*26*) under preindustrial climate conditions with a fixed atmospheric partial pressure of CO_2_ (pCO_2_) (see Materials and Methods). To elucidate the linkage between pN:P and dN:P, we then perturb phyN:P by varying the subsistence N and P quotas and evaluate the resulting changes in dN:P and pN:P in a factorial sensitivity analysis. Our UVic-OPEM reference simulation describes spatial patterns of marine particulate and dissolved elemental stoichiometry, as shown earlier (*14*, *15*). The subsistence quotas largely characterize different functional types (*27*, *28*), so that different subsistence quotas can be viewed as representing the phytoplankton community by different “average” functional types, resulting in different stoichiometry patterns in the world ocean. Thus, we can investigate feedbacks between phyN:P and dN:P and the potential coupling and interdependence between pN:P and dN:P.

Our sensitivity analysis comprises an ensemble of 400 parameter sets with different combinations (20 by 20) of the N and P subsistence quotas of phytoplankton ($Q_{0,\mathrm{phy}}^{N}$, $Q_{0,\mathrm{phy}}^{P}$). The ranges of $Q_{0,\mathrm{phy}}^{N}$ and $Q_{0,\mathrm{phy}}^{P}$ extend by factors between about 3.5 and 5 around the values of the reference simulation, yielding $Q_{0,\mathrm{phy}}^{N}$:$Q_{0,\mathrm{phy}}^{P}$ ratios between 1 and 400 mol mol^−1^. Atmospheric pCO_2_ is set to evolve freely to analyze potential impacts of variations in $Q_{0,\mathrm{phy}}^{N}$ and $Q_{0,\mathrm{phy}}^{P}$ on the global C cycle. All other settings and parameters are left unchanged from the reference simulation, including those of the diazotrophs (table S1). Other factors that can affect phytoplankton community structure and the biogeochemical cycles of N and P, such as the source of inorganic N (*29*, *30*) and dissolved organic P (*31*, *32*), are beyond the scope of our analysis, because they are not part of OPEM.

## RESULTS

### Oceanic pN:C, pP:C, and pN:P in UVic-OPEM

**Fig. 1.**
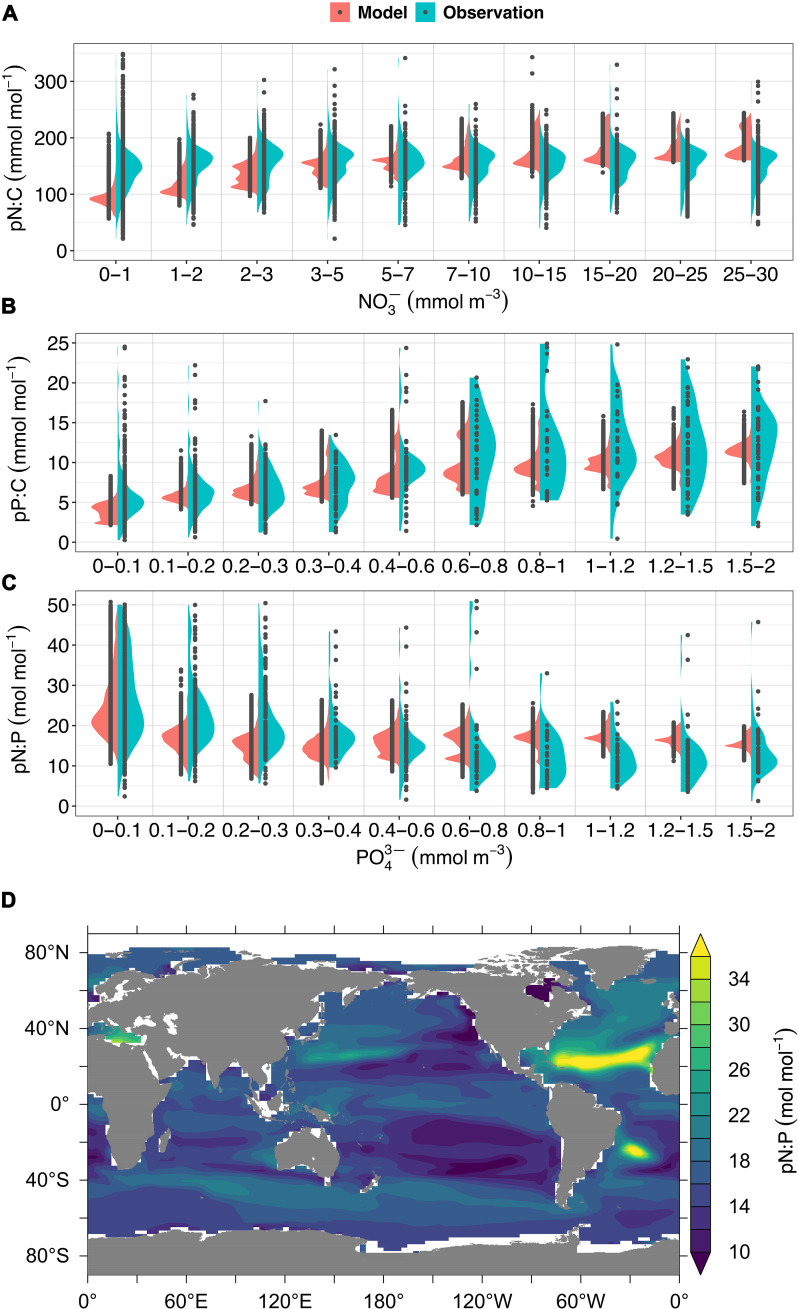
Elemental stoichiometry of surface (0 to 50 m) particulate organic matter (POM). (**A** to **C**) Comparison of the reference simulation of pN:C, pP:C, and pN:P ratios with observed values. (**D**) Global map of pN:P. The widths of the green and red areas in (A) to (C) indicate the probability densities within the concentration ranges of ${\mathrm{NO}}_{3}^{-}$ and ${\mathrm{PO}}_{4}^{3-}$ depicted on the *x* axes. Note that the bin widths are finer at low concentrations. The observations contain 12, 25, and 67 outliers not shown in the panels among the 6789, 1098, and 1146 data points for pN:C, pP:C, and pN:P, respectively. Model results from ice-covered ocean areas are excluded. The observational data were remapped to a 1° × 1° grid with 10 depth levels. Median values have been applied where multiple data points occur in the same grid cell in the same month, and the corresponding ${\mathrm{PO}}_{4}^{3-}$ and ${\mathrm{NO}}_{3}^{-}$ concentrations are from the World Ocean Atlas 2013 (*26*).

The surface (0 to 50 m) POM stoichiometry in the calibrated reference simulation is assessed against observations (*33*) in Fig. 1. Albeit less variable, simulated pN:C and pP:C largely lie within the ranges of the observations (Fig. 1, A and B). The pN:C ratios are slightly underestimated at low ${\mathrm{NO}}_{3}^{-}$ concentrations (≤3 mmol m^−3^), possibly because of the utilization of bioavailable dissolved organic N by phytoplankton under N-limiting conditions (*34*), a process unresolved in the model. The highest values of simulated pN:P are found in regions where ${\mathrm{PO}}_{4}^{3-}$ is scarce and pP:C is lowest, e.g., in the subtropical North Atlantic (Fig. 1, C and D).

### The linkage between pN:P and dN:P

**Fig. 2.**
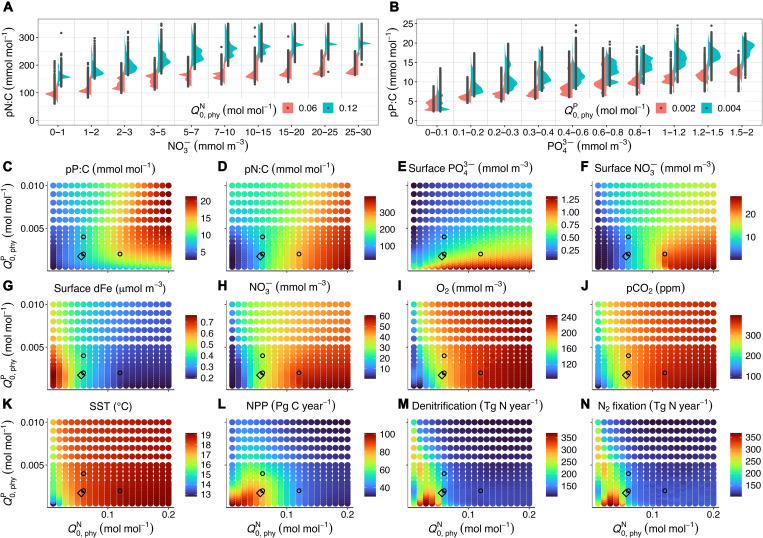
Biogeochemical status as a function of $Q_{0,phy}^{N}$ and $Q_{0,phy}^{P}$. (**A**) Comparison of surface pN:C for $Q_{0,\mathrm{phy}}^{N}=0.06$ and 0.12 mol mol^−1^ at $Q_{0,\mathrm{phy}}^{P}=2\;\mathrm{mmol}\;\mathrm{mo}l^{-1}$. (**B**) Comparison of surface pP:C in two model configurations with $Q_{0,\mathrm{phy}}^{P}=2$ and 4 mmol mol^−1^ at $Q_{0,\mathrm{phy}}^{N}=0.06\;{\text{mol mol}}^{-1}$. (**C** to **N**) pP:C, pN:C, surface ${\mathrm{PO}}_{4}^{3-}$, surface ${\mathrm{NO}}_{3}^{-}$, surface dFe, globally averaged ${\mathrm{NO}}_{3}^{-}$, globally averaged O_2_, atmospheric pCO_2_, as well as averaged sea surface temperature (SST), net primary production (NPP), water column plus benthic denitrification (Denitrification), and N_2_ fixation for all 400 ensemble simulations. pP:C and pN:C are median values of the surface data (0 to 50 m), and surface ${\mathrm{PO}}_{4}^{3-}$, surface ${\mathrm{NO}}_{3}^{-}$, and surface dFe are the averaged surface concentrations. NPP, denitrification, and N_2_ fixation are globally integrated. The three black circles in (C) to (N) indicate the three simulations shown in (A) and (B). Note that the spatial distributions of surface ${\mathrm{NO}}_{3}^{-}$ and ${\mathrm{PO}}_{4}^{3-}$ vary between the different simulations; hence, the nutrient bins in (A) and (B) may be associated with different regions. Tg N, teragrams of nitrogen; Pg C, petagrams of carbon.

The subsistence quotas ($Q_{0,\mathrm{phy}}^{N}$ and $Q_{0,\mathrm{phy}}^{P}$) broadly determine the ranges of the realized phytoplankton N and P quotas (phyN:C and phyP:C) in the model. Owing to the large contribution of phytoplankton to total POM in our model (fig. S1), phyN:C, phyP:C, and phyN:P largely determine pN:C, pP:C, and pN:P (cf. Fig. 1 and figs. S2 to S4). For example, at $Q_{0,\mathrm{phy}}^{P}=2$ mmol mol^−1^, a doubling in $Q_{0,\mathrm{phy}}^{N}$ from 0.06 to 0.12 mol mol^−1^ causes median phyN:C and pN:C to rise by about 50% (Fig. 2A and fig. S5A). Global median pN:C even doubles from 0.14 to 0.28 mol mol^−1^ for this change in $Q_{0,\mathrm{phy}}^{N}$ (Fig. 2D).

The sensitivity of the global median pN:C is enhanced by a strong concomitant increase in the fraction of the ocean surface area with high ${\mathrm{NO}}_{3}^{-}$ concentrations, so that the median surface ${\mathrm{NO}}_{3}^{-}$ concentration increases from ≈7 mmol m^−3^ for $Q_{0,\mathrm{phy}}^{N}=0.06$ mol mol^−1^ to ≈21 mmol m^−3^ for $Q_{0,\mathrm{phy}}^{N}=0.12$ mol mol^−1^ (Fig. 2F). Similarly, at $Q_{0,\mathrm{phy}}^{N}=0.06$ mol mol^−1^, a doubling in $Q_{0,\mathrm{phy}}^{P}$ from 2 to 4 mmol mol^−1^ causes an increase by about 50% in phyP:C (fig. S5B) and a rise in pP:C by about 40%, depending on the ambient ${\mathrm{PO}}_{4}^{3-}$ concentration (Fig. 2B).

In contrast to the relation between $Q_{0,\mathrm{phy}}^{N}$ and the global median pN:C, however, the effect of the same increase in $Q_{0,\mathrm{phy}}^{P}$ on the global median pP:C appears rather subdued, amounting to only about 1% (Fig. 2C). This reduced sensitivity in global median pP:C results from a negative feedback between phyP:C and surface ${\mathrm{PO}}_{4}^{3-}$. That is, when $Q_{0,\mathrm{phy}}^{P}$ increases, phyP:C and, hence, the demand for ${\mathrm{PO}}_{4}^{3-}$ increases and phytoplankton takes up more ${\mathrm{PO}}_{4}^{3-}$. Thus, the sea surface area with low ${\mathrm{PO}}_{4}^{3-}$ concentration increases, which reduces the global median pP:C (Fig. 2E).

The divergent responses in simulated pN:C and pP:C to changes in the subsistence quotas are associated with the different controls of dissolved inorganic ${\mathrm{PO}}_{4}^{3-}$ and ${\mathrm{NO}}_{3}^{-}$ inventories. Because the ${\mathrm{PO}}_{4}^{3-}$ inventory is fixed in the model, changes in model parameters only cause a spatial redistribution of ${\mathrm{PO}}_{4}^{3-}$ within the global ocean. By contrast, the ${\mathrm{NO}}_{3}^{-}$ inventory can vary, as it is sensitive to rates of N_2_ fixation and denitrification in the water column and the sediments (Fig. 2, H, M, and N), which contribute to the different sensitivities of pN:C and pP:C to changes in $Q_{0,\mathrm{phy}}^{N}$ and $Q_{0,\mathrm{phy}}^{P}$, respectively.

The subsistence N and P quotas for diazotrophs ($Q_{0,\mathrm{dia}}^{N}$ and $Q_{0,\mathrm{dia}}^{P}$) are fixed in the ensemble simulations, and the ranges of $Q_{0,\mathrm{phy}}^{N}$ and $Q_{0,\mathrm{phy}}^{P}$ encompass $Q_{0,\mathrm{dia}}^{N}$ and $Q_{0,\mathrm{dia}}^{P}$. When $Q_{0,\mathrm{phy}}^{N}$ or $Q_{0,\mathrm{phy}}^{P}$ is high enough, the (facultative) diazotrophs can outcompete phytoplankton even in areas with high surface ${\mathrm{NO}}_{3}^{-}$. Diazotrophs can contribute up to 37% to total surface POM in these cases (fig. S6), although N_2_ fixation always remains restricted to low ${\mathrm{NO}}_{3}^{-}$ conditions. When $Q_{0,\mathrm{phy}}^{P}$ is high, phytoplankton N:P can be as low as 1.3 mol mol^−1^, and diazotroph N:P can be as high as 53 mol mol^−1^, resulting in a globally averaged pN:P of up to 28 (fig. S7, G, H, and J).

## DISCUSSION

### Interdependence between dN:P and pN:P

The global dN:P is most sensitive to changes in $Q_{0,\mathrm{phy}}^{N}$ in our ensemble simulations because of the interplay between diazotrophs and other phytoplankton. The oceanic ${\mathrm{NO}}_{3}^{-}$ inventory is driven by the balance of N_2_ fixation by diazotrophs and denitrification in the water column and sediments under oxygen (O_2_)–deficient conditions. The effect of varying subsistence quotas on the ${\mathrm{NO}}_{3}^{-}$ inventory is best appreciated by combining the effect on N_2_ fixation with that on denitrification, mediated by net primary production (NPP) and its effect on O_2_. Increasing $Q_{0,\mathrm{phy}}^{N}$ raises phyN:P and the consumption of ${\mathrm{NO}}_{3}^{-}$, which increases pN:P but lowers surface ${\mathrm{NO}}_{3}^{-}$ concentrations and, hence, promotes N_2_ fixation. However, the higher N demand combined with lower surface ${\mathrm{NO}}_{3}^{-}$ eventually reduces NPP and the subsequent POM export and remineralisation at depth, which leads to less O_2_ consumption and, thus, less denitrification. Both increased N_2_ fixation and reduced denitrification add up, increasing the ${\mathrm{NO}}_{3}^{-}$ inventory. Decreasing $Q_{0,\mathrm{phy}}^{P}$ increases phyN:P, NPP, and also N_2_ fixation, but rising NPP and POM export also leads to higher O_2_ consumption, which increases denitrification and counteracts the increase in N_2_ fixation. This negative feedback, thus, limits the effect of $Q_{0,\mathrm{phy}}^{P}$ on dN:P.

**Fig. 3.**
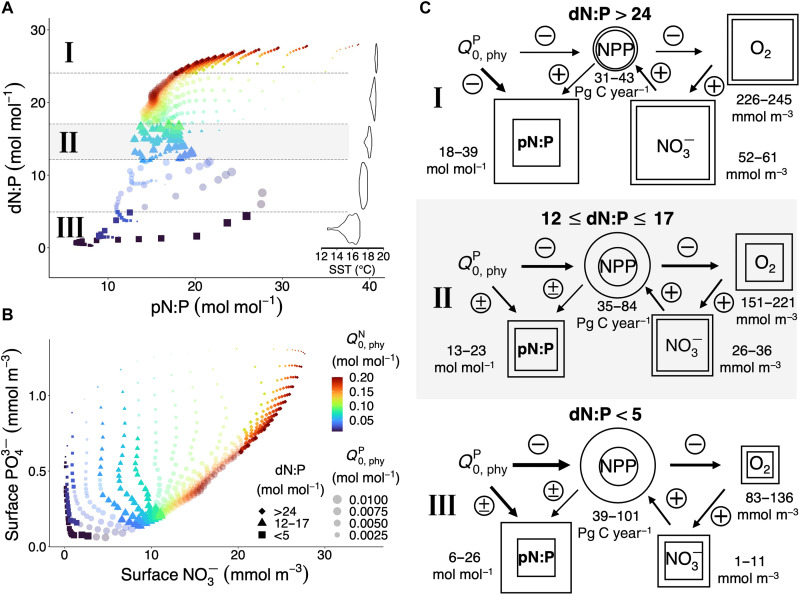
Effects of different $Q_{0,phy}^{N}$ and $Q_{0,phy}^{P}$ on marine biogeochemistry. (**A**) dN:P versus median surface pN:P and global mean sea surface temperature (SST). The dashed lines delineate regions with different dN:P ranges: I (dN:P > 24 mol mol^−1^; diamonds), II (12 < dN:P < 17; triangles), and III (dN:P < 5; squares). (**B**) Surface ${\mathrm{NO}}_{3}^{-}$ versus ${\mathrm{PO}}_{4}^{3-}$. In (A) and (B), symbol color indicates $Q_{0,\mathrm{phy}}^{N}$ and size $Q_{0,\mathrm{phy}}^{P}$. (**C**) Schematic of the biological control and biogeochemical feedbacks for the three dN:P ranges shown in (A). Circles and squares represent ranges of the fluxes and tracer levels, respectively. The relative strength of control for individual components among the three regions is indicated by the arrow thickness. The plus and minus signs specify positive and negative controls, respectively; when both signs are present, both directions of controls exist. NPP, net primary production; Pg C, petagrams of carbon.

While previous studies, assuming constant phyN:P, have concluded that dN:P should be linearly correlated with pN:P (*3*, *4*), this relationship turns out to be more complex in our ensemble of simulations, allowing for variable phytoplankton stoichiometry. dN:P generally does increase with higher pN:P (Fig. 3A), but we identify two notable features: (i) Simulated global average pN:P and dN:P do not form a one-to-one (injective) functional relation across our ensemble of simulated worlds, differing only in phytoplankton subsistence quotas. Instead, it allows for similar dN:P being attributable to different pN:P and vice versa (Fig. 3A), and (ii) dN:P has an upper limit of around 28 in our ensemble simulations, although pN:P can increase further. The deviant, nonlinear relationship between pN:P and dN:P can be attributed to differences in spatiotemporal variations between phyN:C and phyP:C, because they exert indirect but strong control on dN:P via O_2_ (Figs. 2I and 3C).

A negative feedback regulating the marine nitrogen inventory has been described previously in a qualitative manner, where O_2_ acts as a biogeochemical mediator in the ocean that connects variations in pN:P to variations in dN:P (*35*, *36*). Variations in O_2_ concentration reflect variations in primary production and the ensuing export and respiration, which, in turn, is influenced by changes in phyN:C and phyP:C (Fig. 2I). Once O_2_ is depleted (O_2_-deficient zones), denitrification sets in, reducing the ${\mathrm{NO}}_{3}^{-}$ inventory and, hence, dN:P. In turn, changes in dN:P affect the rate of N_2_ fixation of the diazotrophs, which compensates for ${\mathrm{NO}}_{3}^{-}$ losses from denitrification once a steady state is reached and a different ${\mathrm{NO}}_{3}^{-}$ inventory is established.

A key finding is that contrary to dN:P, which is driven mainly by changes in $Q_{0,\mathrm{phy}}^{N}$, the main driver of the variations in pN:P is $Q_{0,\mathrm{phy}}^{P}$. The range of realized pN:P among the simulations is narrowest when dN:P is close to the Redfield ratio (region II in Fig. 3), owing to an effective negative biogeochemical feedback between variations in ${\mathrm{PO}}_{4}^{3-}$ and pN:P: Increasing $Q_{0,\mathrm{phy}}^{P}$ raises the P demand for N assimilation in phytoplankton, leading to a decline in both phyN:P (fig. S7G) and surface ${\mathrm{PO}}_{4}^{3-}$ concentrations (Fig. 2E). This results in a competitive advantage for diazotrophs, which, owing to the high diazotroph’s N:P ratio, can counteract the lower phyN:P and thereby reduce the variability of pN:P. Under these conditions, NPP is co-limited by N and P.

This negative feedback is less effective in both the low and the high dN:P ranges (regions I and III in Fig. 3), however, where $Q_{0,\mathrm{phy}}^{P}$ exerts much stronger control on pN:P, albeit for different reasons. In the high dN:P range (region I in Fig. 3A), NPP is mostly Fe limited, and relatively high surface macronutrient concentrations (Fig. 3B) largely diminish the diazotrophs’ advantage. Hence, diazotrophs have little effect on NPP and O_2_ and cannot compensate for changes in phyN:P. In contrast, owing to the low ${\mathrm{NO}}_{3}^{-}$ conditions associated with the lower dN:P range (region III in Fig. 3B), variations in pN:P are mostly driven by changes in diazotroph abundance benefitting from high $Q_{0,\mathrm{phy}}^{P}$ (fig. S6B), also shifting the ocean from ${\mathrm{NO}}_{3}^{-}$ to ${\mathrm{PO}}_{4}^{3-}$ limitation as $Q_{0,\mathrm{phy}}^{P}$ increases (Fig. 3B). As a result, pN:P, global NPP, O_2_, and ${\mathrm{NO}}_{3}^{-}$ are all highly sensitive to changes in $Q_{0,\mathrm{phy}}^{P}$ in the lower dN:P range (region III in Fig. 3, A and C). The increase in relative diazotroph abundance with increasing $Q_{0,\mathrm{phy}}^{P}$, together with the low $Q_{0,\mathrm{phy}}^{N}$, also causes a positive relation between pN:P and $Q_{0,\mathrm{phy}}^{P}$ in this region, which is opposite to that in region I. For dN:P close to the Redfield ratio (region II in Fig. 3), pN:P is less variable because of the negative feedback between ${\mathrm{PO}}_{4}^{3-}$ and pP:C, which is most effective when NPP is co-limited by N and P.

### Biogeochemical constraints on the changes in dN:P and pN:P

**Fig. 4.**
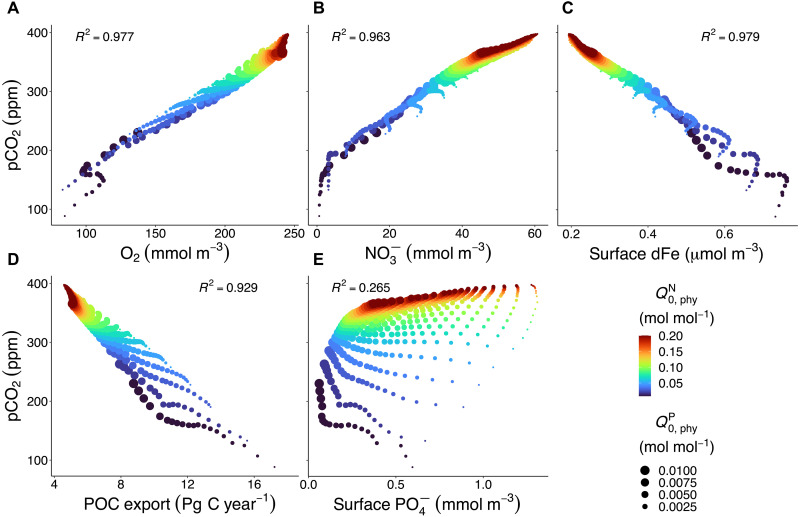
Effects of different $Q_{0,phy}^{N}$ and $Q_{0,phy}^{P}$ on marine biogeochemistry and atmospheric pCO_2_ in our ensemble of simulations. (**A** to **E**) Atmospheric partial pressure of CO2 (pCO_2_) versus globally averaged marine O_2_, ${\mathrm{NO}}_{3}^{-}$, surface dFe, globally integrated particulate organic carbon (POC) export at 130 m, and surface ${\mathrm{PO}}_{4}^{3-}$. *R*^2^, square of the Pearson product moment correlation coefficient; *n* = 400. Pg C, petagrams of carbon.

Two feedbacks restrict dN:P to values below ≈28 for high $Q_{0,\mathrm{phy}}^{N}$, in spite of the low denitrification associated with low primary production and low particulate organic carbon (POC) export found at the upper end of the dN:P range (Fig. 4D). (i) High dN:P suppresses N_2_ fixation, because the facultative diazotrophs in UVic-OPEM switch to using ${\mathrm{NO}}_{3}^{-}$ at high ambient ${\mathrm{NO}}_{3}^{-}$ concentrations. (ii) Dissolved iron (dFe) limits phytoplankton photosynthesis and N assimilation in UVic-OPEM, resulting in an inverse relation between surface dFe and ${\mathrm{NO}}_{3}^{-}$ concentrations (Fig. 2, G and H). Because increasing $Q_{0,\mathrm{phy}}^{N}$ increases phytoplankton Fe demand, surface dFe decreases toward high $Q_{0,\mathrm{phy}}^{N}$, resulting in more severe Fe limitation and higher ${\mathrm{NO}}_{3}^{-}$ concentrations. Thus, Fe limitation of N_2_ fixation also contributes to limiting dN:P, in spite of the low denitrification associated with the low NPP and POC export at high $Q_{0,\mathrm{phy}}^{N}$. Together, these constitute a negative feedback between surface ${\mathrm{NO}}_{3}^{-}$ and N_2_ fixation, which limits the maximum achievable surface ${\mathrm{NO}}_{3}^{-}$ concentration.

Nitrate concentrations at the lower end of the dN:P range can be very low because of high rates of denitrification associated with the low O_2_ content of the ocean under these conditions. N_2_ fixation often cannot keep up with denitrification, particularly if both processes become spatially coupled. The strength of the coupling between N_2_ fixation and denitrification is sensitive to the ecological niche of the diazotrophs, i.e., assumptions made with respect to the drivers of N_2_ fixation (*37*).

Two major negative feedbacks limit the changes in pN:P at the global scale in our ensemble of simulations: (i) The negative feedback between phyP:C and surface ${\mathrm{PO}}_{4}^{3-}$ restricts the ranges of pP:C and pN:P, and (ii) the presence of diazotrophs prevents pN:P from getting as low as that of phytoplankton at high $Q_{0,\mathrm{phy}}^{P}$ and low $Q_{0,\mathrm{phy}}^{N}$. The diazotrophs also limit the increase of pN:P for high $Q_{0,\mathrm{phy}}^{N}$ and low $Q_{0,\mathrm{phy}}^{P}$ (fig. S7). These negative feedbacks result in pN:P staying between 6 and 38 mol mol^−1^ (Fig. 3A), although $Q_{0,\mathrm{phy}}^{N}$:$Q_{0,\mathrm{phy}}^{P}$ ranges from 1 to 400 mol mol^−1^.

### Biotic controls on marine O_2_ and atmospheric pCO_2_

The growth of phytoplankton and diazotrophs determines the rate of CO_2_ fixation, which reduces atmospheric pCO_2_ due to enhanced air-sea gas flux of CO_2_ (Fig. 2J). We find a strong correlation between atmospheric pCO_2_ and the oceanic O_2_ inventory (*R*^2^ = 0.98; Fig. 4A). A large oceanic O_2_ inventory corresponds to low remineralization and, thus, low oceanic C storage and high atmospheric pCO_2_. A small oceanic O_2_ inventory corresponds to high remineralization and high oceanic C storage, leaving less CO_2_ in the atmosphere. Because O_2_ is the main mediator between the inventories of C and N, the strong correlation between atmospheric pCO_2_ and the oceanic O_2_ inventory implies a mutual dependence between atmospheric pCO_2_ and the marine ${\mathrm{NO}}_{3}^{-}$ inventory. pCO_2_ and ${\mathrm{NO}}_{3}^{-}$ are highly correlated (*R*^2^ = 0.96) across our ensemble simulations (Fig. 4B). Thus, the eventual imprint of $Q_{0,\mathrm{phy}}^{N}$ and $Q_{0,\mathrm{phy}}^{P}$ on the ${\mathrm{NO}}_{3}^{-}$ inventory in the model translates into a concomitant, roughly proportional change in atmospheric pCO_2_, which affects the climate and ocean circulation in the model. Because surface dFe largely limits the marine C fixation, atmospheric pCO_2_ and surface dFe are highly negatively correlated (*R*^2^ = 0.98; Fig. 4C).

In the ensemble simulations, the POC export is negatively correlated with the pCO_2_ (Fig. 4D), and the POC export is as high as 17 petagrams of carbon (Pg C) per year when the pCO_2_ is down to 88 parts per million (ppm) and is as low as 4.6 Pg C per year when the pCO_2_ is close to 400 ppm. Surface ${\mathrm{PO}}_{4}^{3-}$ concentration has been used as an indicator for atmospheric pCO_2_ because the utilization of surface ${\mathrm{PO}}_{4}^{3-}$ is associated with C fixation (*38*–*40*). This relation becomes more complicated when considering a variable pP:C, which may amplify (*38*, *41*) or damp (*23*) the effect of surface ${\mathrm{PO}}_{4}^{3-}$ changes on atmospheric pCO_2_. In the present study, the correlation between pCO_2_ and surface ${\mathrm{PO}}_{4}^{3-}$ depends on whether we are looking at variable $Q_{0,\mathrm{phy}}^{N}$ or $Q_{0,\mathrm{phy}}^{P}$. For constant $Q_{0,\mathrm{phy}}^{P}$, the correlation is mostly positive, as both NPP and ${\mathrm{PO}}_{4}^{3-}$ decline and, hence, both pCO_2_ and surface ${\mathrm{PO}}_{4}^{3-}$ rise with increasing $Q_{0,\mathrm{phy}}^{N}$. However, for constant $Q_{0,\mathrm{phy}}^{N}$, the correlation ranges from negative for small $Q_{0,\mathrm{phy}}^{N}$, associated with region III in Fig. 3A, to weakly positive for large $Q_{0,\mathrm{phy}}^{N}$, associated with region I in Fig. 3A (Fig. 4E). In contrast to ${\mathrm{NO}}_{3}^{-}$ (Fig. 4B), no clear relation emerges between pCO_2_ and surface ${\mathrm{PO}}_{4}^{3-}$ among the whole ensemble of simulations (Fig. 4E). Thus, varying phytoplankton subsistence quotas in UVic-OPEM break the relationship between pCO_2_ and surface ${\mathrm{PO}}_{4}^{3-}$ postulated in previous studies (*38*–*40*).

### Climate constraints on the N:P of phytoplankton

**Fig. 5.**
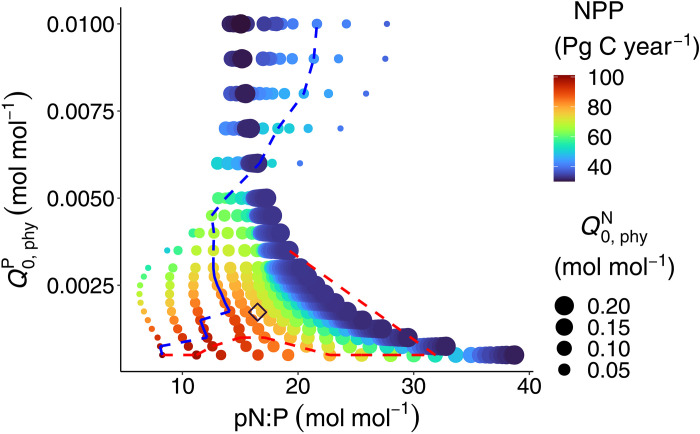
Net primary production (NPP) as a function of pN:P (horizontal axis) and $Q_{0,phy}^{P}$ (vertical axis). The blue dashed line indicates the maximum NPP at each given $Q_{0,\mathrm{phy}}^{P}$, and the red dashed line denotes the maximum NPP at each given $Q_{0,\mathrm{phy}}^{N}$. The open diamond shows the position of the reference simulation. Pg C, petagrams of carbon.

NPP in our ensemble is maximal for a specific range of optimal combinations of $Q_{0,\mathrm{phy}}^{N}$ and $Q_{0,\mathrm{phy}}^{P}$. Subsistence quotas may be viewed as costs to phytoplankton growth, also because of the higher Fe demand when $Q_{0,\mathrm{phy}}^{N}$ increases. Thus, it is not unexpected that the maximum globally integrated NPP occurs in the simulation with the lowest $Q_{0,\mathrm{phy}}^{N}$ and $Q_{0,\mathrm{phy}}^{P}$ (Fig. 2L). Nevertheless, the low pCO_2_ in simulations with very low subsistence quotas lowers sea surface temperature (SST; Fig. 2K) and, hence, also NPP. For a given $Q_{0,\mathrm{phy}}^{P}$, the maximum NPP does not occur at the lowest $Q_{0,\mathrm{phy}}^{N}$, when $Q_{0,\mathrm{phy}}^{P}>0.001\;{\text{mol mol}}^{-1}$ (blue dashed line, Fig. 5), and for a given $Q_{0,\mathrm{phy}}^{N}$, the $Q_{0,\mathrm{phy}}^{P}$ that yields the maximum NPP is not the lowest when $Q_{0,\mathrm{phy}}^{N}\approx 0.04\;{\text{mol mol}}^{-1}$ (red dashed line, Fig. 5). Notably, the combination of $Q_{0,\mathrm{phy}}^{N}$ and $Q_{0,\mathrm{phy}}^{P}$ of our reference solution, which was calibrated against global data with a median dN:P close to the Redfield ratio, lies in the vicinity of the maximum NPP for $Q_{0,\mathrm{phy}}^{N}\approx 0.04\;{\text{mol mol}}^{-1}$ (open diamond, Fig. 5).

### Implication for the role of phytoplankton physiology in marine nutrient cycling and global climate

Our simulations encompass a very wide spread in pCO_2_ (≈100 to >350 ppm; Fig. 2J), which, in this case, obviously originates from differences in the biological soft tissue pump (*42*), as phytoplankton is the only model component directly affected by the subsistence quotas. This may appear unexpected because changes in the biological pump usually are considered a minor contribution to variations in ocean C storage in the current climate change context, albeit with widely differing estimates (*43*, *44*). Analyses of anthropogenic CO_2_ uptake by the ocean concern transient behavior on a time scale of decades to centuries, however, whereas our results refer to equilibrium Earth system states on a time scale of 10^4^ years. In addition, wide-spread air-sea CO_2_ disequilibria have recently been shown to amplify effects of a changed biological pumping to such an extent that it could be considered a major contributor to the glacial-interglacial pCO_2_ difference (*45*).

Our model results demonstrate the importance of considering variable stoichiometry when analyzing the response of marine biogeochemistry to phytoplankton physiological variations. Therefore, the negative feedbacks stabilizing the marine nitrogen cycle in our study differ fundamentally from the “nutrient thermostat” described in earlier studies (*2*, *17*), where an assumed fixed average pN:P ratio acts as a trigger for N_2_ fixation. Allowing for local deviations from the fixed pN:P ratio also can have considerable effects on dN:P (*17*). However, the nutrient thermostat concept would essentially imply a tight spatial association of N_2_ fixation and O_2_ minimum zones (*46*), which could result in a positive feedback between N_2_ fixation and denitrification (*47*) and, thus, render this mechanism ineffective for stabilizing the marine nitrogen cycle (*48*). Our results further show that it is critical to consider also variations in C to nutrient ratios, which largely affect the growth of phytoplankton and denitrification, the major loss of nitrate in the ocean. Descriptions of the physiology of tiny plankton organisms can help elucidate profound interdependencies between particulate organic and dissolved inorganic matter pools in the global ocean. Accordingly, laboratory and field experiments that focus on physiological details, such as subsistence quotas of diverse phytoplankton, are not only valuable for explaining plankton dynamics but also of relevance for global marine biogeochemistry and climate.

## MATERIALS AND METHODS

### Model description

For our study, we apply the UVic-ESCM (*16*) coupled to the OPEM (*14*, *15*), referred to as UVic-OPEM, in the following. The UVic-ESCM contains three components, a terrestrial model, a one-layer energy-moisture balance atmosphere model, and a three-dimensional ocean general circulation model. The horizontal resolution of the ocean, land, and atmosphere components is 3.6° × 1.8°, and the ocean has 19 vertical layers. UVic-ESCM is widely used as a comprehensive global C cycle model for simulations under various climate settings (*49*–*51*) and has also participated in the Climate Model Intercomparison Project 6 (*52*).

OPEM comprises four POM pools: non–N_2_-fixing phytoplankton (phytoplankton in the following), diazotrophs, zooplankton, and detritus. The growth of phytoplankton and diazotrophs follows an optimality assumption, where dynamic resource allocation between light-harvesting, ${\mathrm{PO}}_{4}^{3-}$ and ${\mathrm{NO}}_{3}^{-}$ uptake, and N_2_ fixation of diazotrophs maximizes growth rate under changing environmental conditions (light, temperature, and nutrient concentrations) (*28*).

Bioavailable Fe is explicitly resolved, and primary production can become limited by the ambient Fe concentration. This limitation is introduced as a Monod equation, with different half-saturation Fe concentrations for phytoplankton and diazotrophs.

Phytoplankton nutrient uptake and CO_2_ fixation follow the temperature function in (*53*). Diazotrophs facultatively fix N_2_, i.e., they use ${\mathrm{NO}}_{3}^{-}$ when N_2_ fixation would yield a lower net growth rate. The formulation of zooplankton foraging is the optimal current feeding model (*54*). The only sinking compartment is the detritus, which is fed by phytoplankton, diazotrophs, and zooplankton mortality, as well as sloppy feeding and egestion of zooplankton. The elemental composition is dynamic in phytoplankton, diazotrophs, and detritus, while the zooplankton has a fixed C:N:P ratio of 106:16:1. Details of the model description and evaluation have been reported in previous articles (*14*, *15*). For the current study, the code has been updated to UVic-ESCM version 2.10 (*52*), which includes the consideration of benthic denitrification (*55*), and we follow the original UVic temperature function for diazotrophs.

### Calibration of the reference simulation

For ecosystem parameter calibration, we conducted 600 simulations under preindustrial conditions with parameter sets constructed with the Latin hypercube (LHC) method (*14*). The LHC sampling has been preferred over the estimation of parameter values with an iterative optimization algorithm here, mainly for two reasons: (i) All 600 simulations can be done in parallel, which reduces computational time considerably, and (ii) the applied metric can be refined and then applied to the existing ensemble without the need for extra model optimizations. Every ensemble member has been spun up for 10,000 years under preindustrial climate conditions with a prescribed atmospheric CO_2_ concentration of 284.3 ppm, achieving approximate steady-state conditions. The reference parameter set (table S1) produces the lowest data model misfit, according to the minimum of a likelihood-based cost function (*14*). In this study, we apply a refined version of the cost function, which still considers surface chlorophyll *a* and ${\mathrm{PO}}_{4}^{3-}$, but ${\mathrm{NO}}_{3}^{-}$ and O_2_ are replaced by $N^{*}=NO_{3}^{-}-16\cdot PO_{4}^{3-}+2.9\;\mathrm{mmol}\;m^{-3}$ and modified apparent oxygen utilization (AOU), ${\mathrm{AOU}}^{*}=\mathrm{AOU}-2\cdot PO_{4}^{3-}$, respectively. ${\mathrm{NO}}_{3}^{-}$, ${\mathrm{PO}}_{4}^{3-}$, and O_2_ data are from the World Ocean Atlas 2013 (*26*).

### Setup of the sensitivity analysis

In OPEM, phytoplankton N and P cell quotas [phyN:C in (molN)(molC)^−1^ and phyP:C in (molP)(molC)^−1^] are allocated to maintenance and growth. The maintenance of a baseline physiology is associated with the subsistence quotas ($Q_{0,\mathrm{phy}}^{N}$ and $Q_{0,\mathrm{phy}}^{P}$), while the remainders ($\mathrm{phyN}:C-Q_{0,\mathrm{phy}}^{N}$ and $\mathrm{phyP}:C-Q_{0,\mathrm{phy}}^{P}$) are allocated to nutrient uptake, biosynthesis, and photosynthesis (*28*). Variations in resource allocation are the primary cause of the plasticity of phytoplankton stoichiometry in our model. Corresponding allocation factors are determined by the optimal growth strategy, and they reflect the acclimation state of phytoplankton. The subsistence N quota $Q_{0,\mathrm{phy}}^{N}$ is proportional to the demands for structural material, and $Q_{0,\mathrm{phy}}^{P}$ represents the P bound in the nucleus and membranes. $Q_{0,\mathrm{phy}}^{N}$ and $Q_{0,\mathrm{phy}}^{P}$ are treated as global constants in UVic-OPEM. Because the parameter values of the non–N_2_-fixing phytoplankton are assumed to be representative means of the entire community, any change of the subsistence quotas ($Q_{0,\mathrm{phy}}^{N}$ and $Q_{0,\mathrm{phy}}^{P}$) can be viewed as a shift in community composition.

In the sensitivity analysis, we vary the two subsistence quotas by specifying an ensemble of 400 (20 × 20) combinations of $Q_{0,\mathrm{phy}}^{N}\in [10^{-2},2\;\cdot 10^{-1}]$ and $Q_{0,\mathrm{phy}}^{P}\in [5\;\cdot 10^{-4},10^{-2}]$. $Q_{0,\mathrm{phy}}^{N}$ varies with an incremental step size $\text{Δ}Q_{0,\mathrm{phy}}^{N}=10^{-2}$. $Q_{0,\mathrm{phy}}^{P}$ varies with incremental step sizes $\text{Δ}Q_{0,\mathrm{phy}}^{P}=2.5\;\cdot 10^{-4}$ for $Q_{0,\mathrm{phy}}^{P}\in [5\;\cdot 10^{-4},3\;\cdot 10^{-3}]$, $\text{Δ}Q_{0,\mathrm{phy}}^{P}=5\;\cdot 10^{-4}$ for $Q_{0,\mathrm{phy}}^{P}\in ]3\;\cdot 10^{-3},5\;\cdot 10^{-3}]$, and $\text{Δ}Q_{0,\mathrm{phy}}^{P}=10^{-3}$ for $Q_{0,\mathrm{phy}}^{P}\in ]5\;\cdot 10^{-3},10^{-2}]$.

For every ensemble member, we start the simulation from the final state of the reference model solution and integrate for another 10,000 years. Apart from a freely evolving atmospheric CO_2_, to analyze possible relations between changes in phytoplankton stoichiometry and pCO_2_, all other parameter values and settings remain identical to the reference simulation. We evaluate the results of the ensemble simulations at year 10,000, when the marine biogeochemical cycles are again close to equilibrium. Steady-state conditions are assessed by the average relative change in the oceanic ${\mathrm{NO}}_{3}^{-}$ inventory over the last 100 years, which ranges from −0.0015 to 0.0043% year^−1^ among the 400 simulations.
