# Supplementary material for: Effects of phytoplankton physiology on global ocean biogeochemistry and climate
Source: Sci Adv. 2023 Jul 26;9(30):eadg1725. doi: 10.1126/sciadv.adg1725 (PMC10371029; doi:10.1126/sciadv.adg1725)
Supplement: Supplementary file 1 — Figs. S1 to S7 Table S1 [file sciadv.adg1725_sm.pdf]

Science Advances
AAAS

Supplementary Materials for

Effects of phytoplankton physiology on global ocean biogeochemistry and climate

Chia-Te Chien *et al.*

Corresponding author: Chia-Te Chien, cchien@geomar.de

Sci. Adv. **9**, eadg1725 (2023)
DOI: 10.1126/sciadv.adg1725

This PDF file includes:

Figs. S1 to S7
Table S1

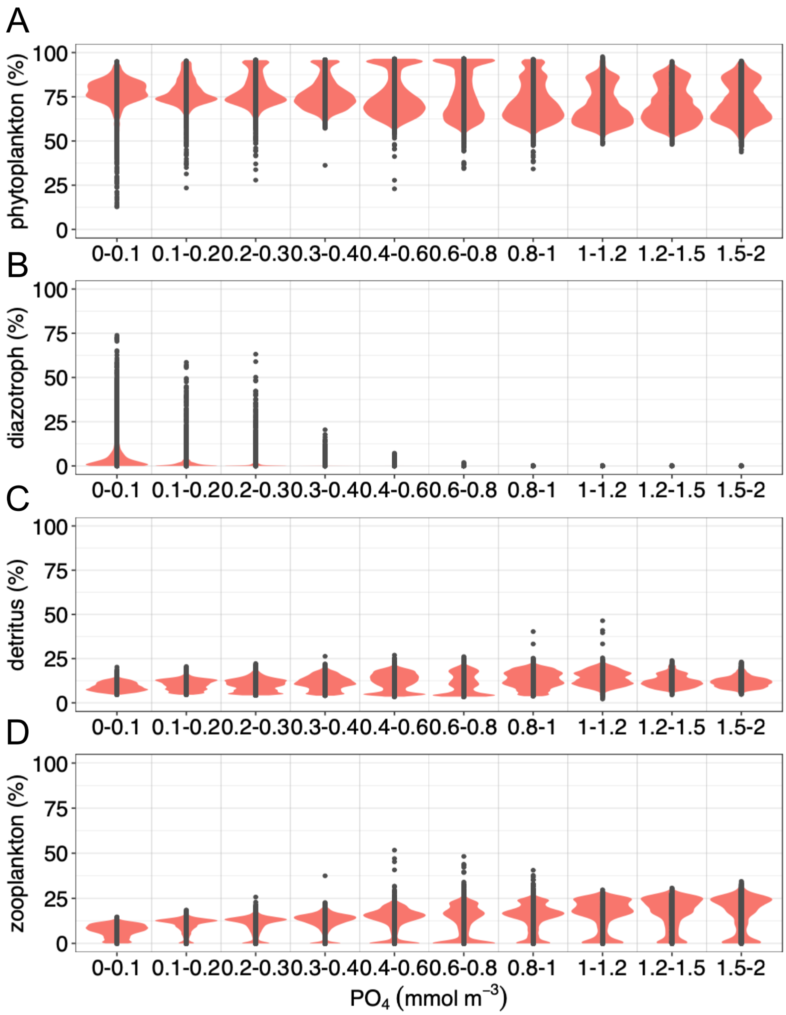

Fig. S1. Relative contributions of (A) phytoplankton, (B) diazotrophs, (C) detritus, and (D) zooplankton to total surface (0 – 50m) POM (carbon) in the reference simulation.

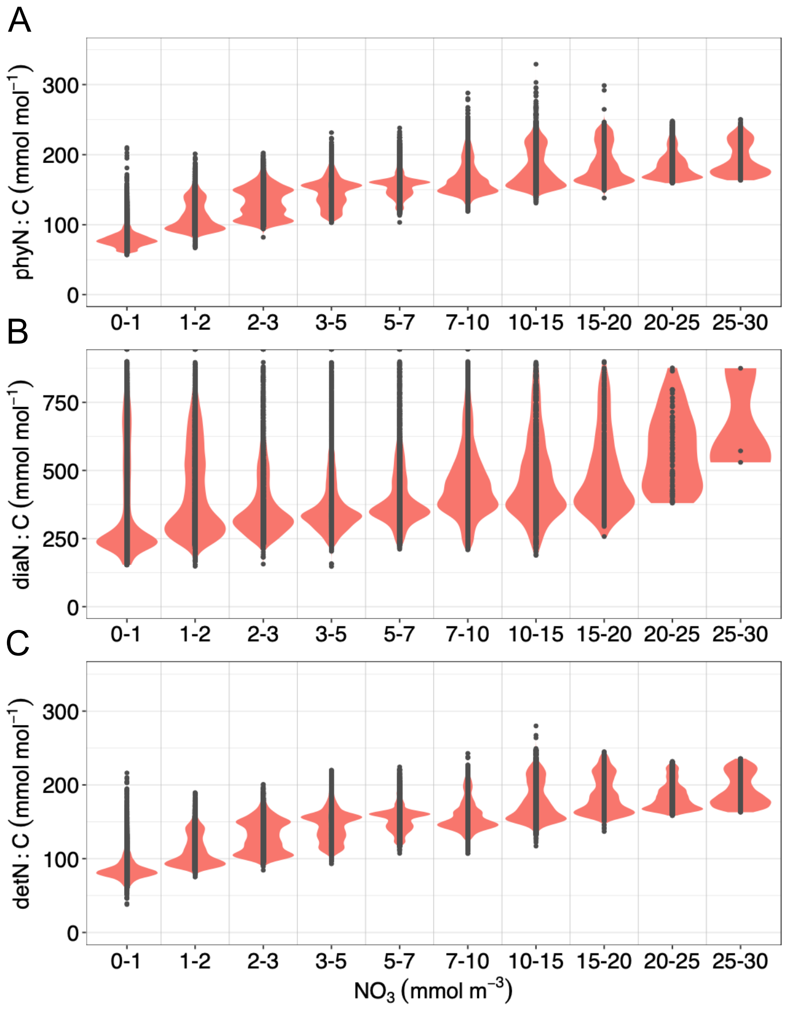

Fig. S2. N:C of (A) (non-N_2 fixing) phytoplankton, (B) diazotrophs, and (C) detritus in the surface layer (0 – 50m) of the reference simulation.

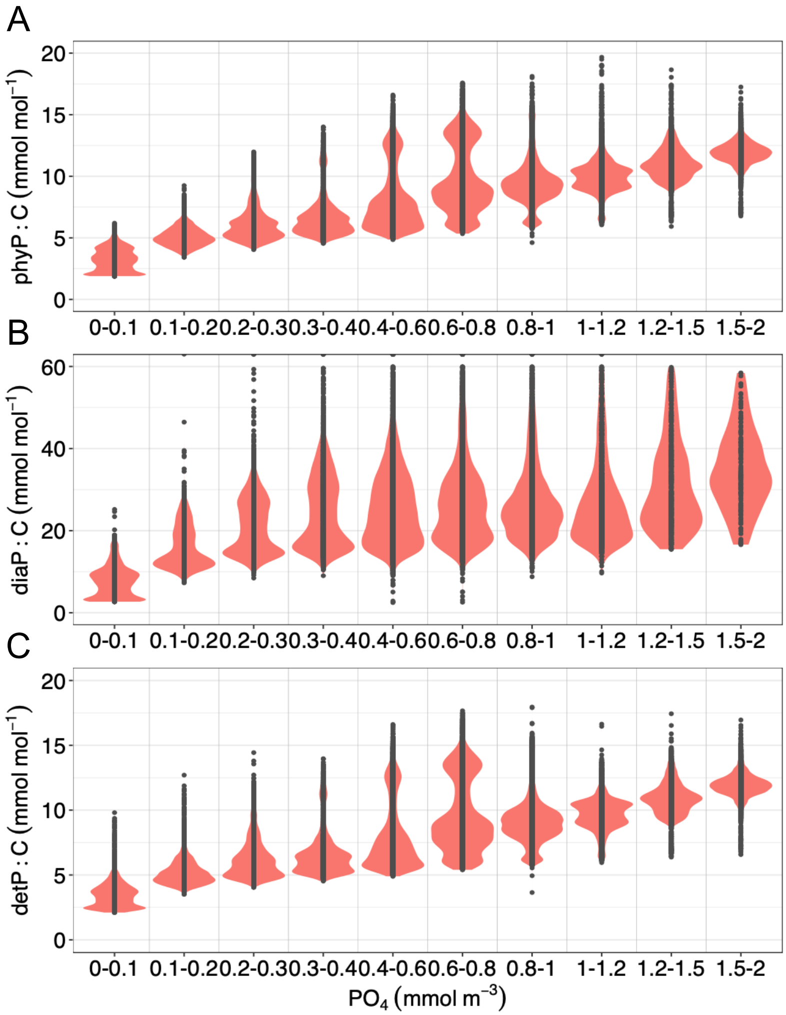

Fig. S3. P:C of (A) phytoplankton, (B) diazotrophs, and (C) detritus in the surface layer (0 – 50m) of the reference simulation.

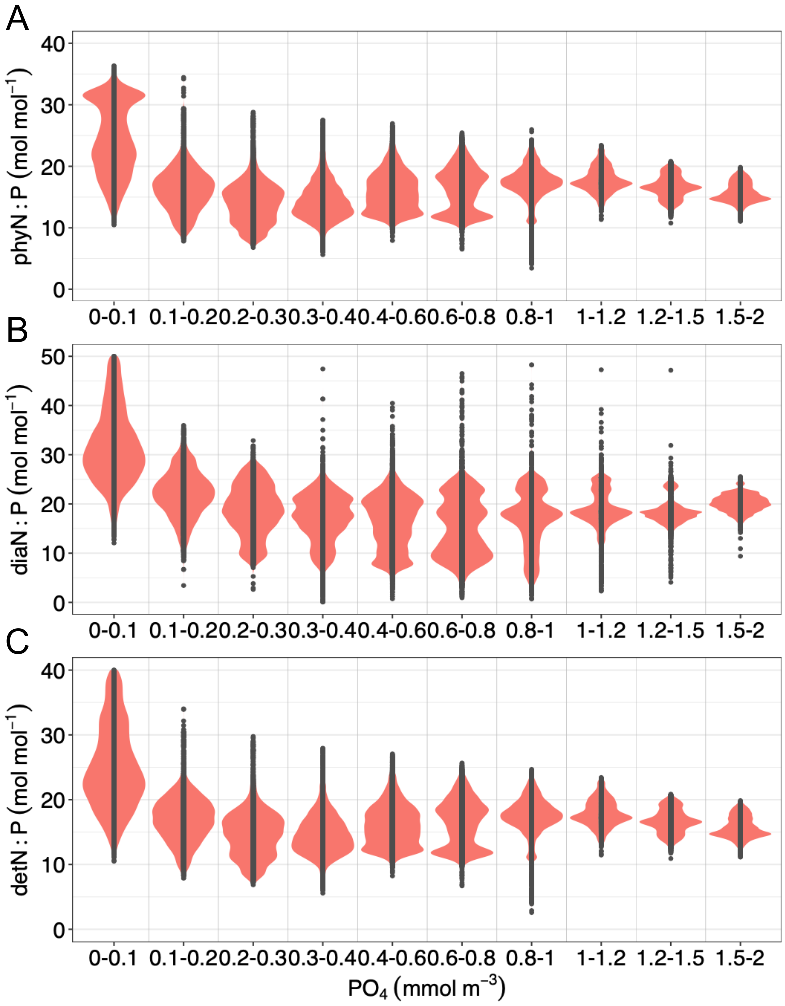

Fig. S4. N:P of (A) phytoplankton, (B) diazotrophs, and (C) detritus in the surface layer (0 – 50m) of the reference simulation.

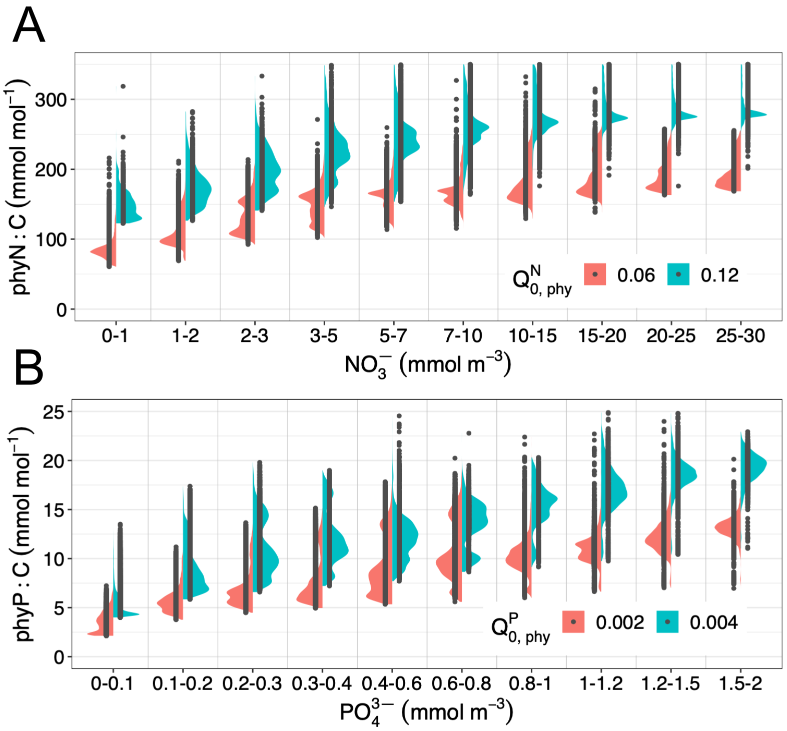

Fig. S5. Comparison of surface phytoplankton stoichiometry in models with different $Q^{N}_{0,\,phy}$ and $Q^{P}_{0,\,phy}$. (**A**) Comparison of surface phytoplankton N:C in two models with $Q^{N}_{0,\,phy}$ of 0.06 and 0.12 at $Q^{P}_{0,\,phy} = 0.002$. (**B**) Comparison of surface phytoplankton P:C in two model configurations with $Q^{P}_{0,\,phy}$ of 0.002 and 0.004 at $Q^{N}_{0,\,phy} = 0.06$.

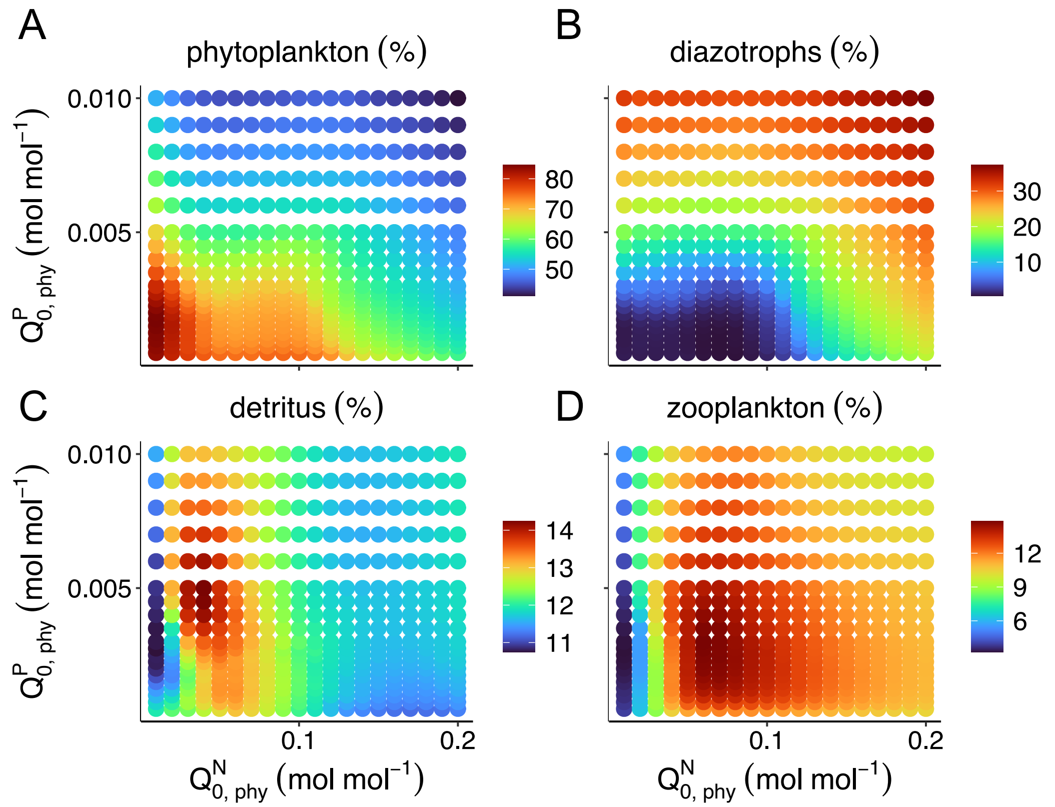

Fig. S6. Globally-integrated (0 – 50m) biomass (carbon) fractions of (A) phytoplankton, (B) diazotrophs, (C) detritus, and (D) zooplankton in all 400 simulations.

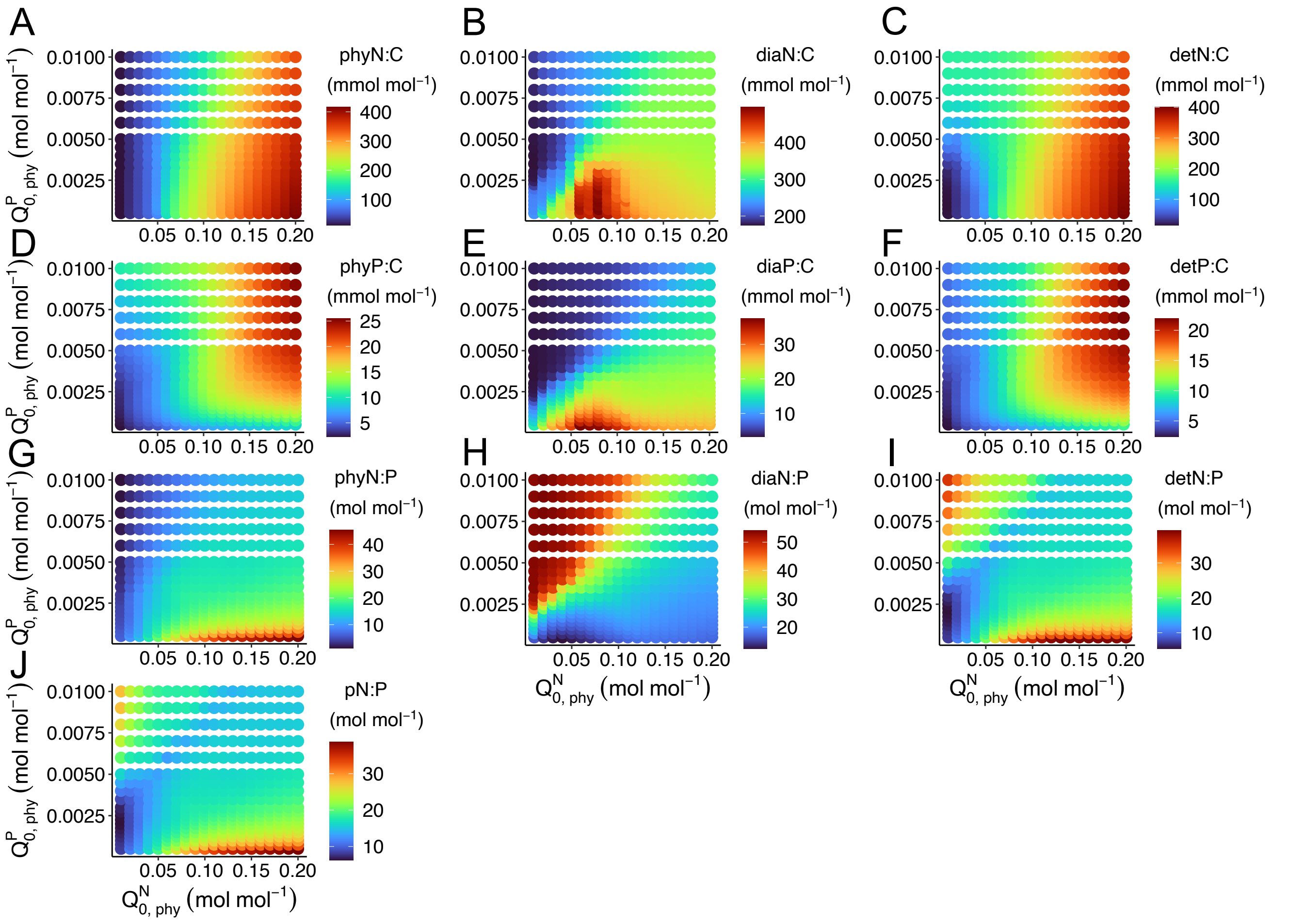

Fig. S7. (A – C) N:C, (D – F) P:C, and (G – I) N:P of phytoplankton, diazotrophs, and detritus, and (J) N:P of POM in all 400 simulations.

Table S1. Names, ranges, reference values, units, and descriptions of the OPEM parameters.

Symbol	Range	Value	Units	Definition
$A_{0,\,\mathrm{phy}}$	216 – 416	344.8397	$\mathrm{m^3\,(mol\,C)^{-1}\,d^{-1}}$	non-N_2 fixing phytoplankton potential nutrient affinity
$A_{0,\,\mathrm{dia}}$	286 – 486	480.6693	$\mathrm{m^3\,(mol\,C)^{-1}\,d^{-1}}$	diazotroph potential nutrient affinity
α_{phy}	0.396 – 0.596	0.54726	$\mathrm{m^3\,(mol\,C)^{-1}\,d^{-1}}$	non-N_2 fixing phytoplankton light affinity
α_{dia}	0.369 – 0.569	0.56464	$\mathrm{m^3\,(mol\,C)^{-1}\,d^{-1}}$	diazotroph light affinity
$Q^{\mathrm{N}}_{0,\,\mathrm{phy}}$	0.056 – 0.064	0.05633	$\mathrm{mol\,(mol\,C)^{-1}}$	non-N_2 fixing phytoplankton subsistence N quota
$Q^{\mathrm{N}}_{0,\,\mathrm{dia}}$	0.12 – 0.15	0.14715	$\mathrm{mol\,(mol\,C)^{-1}}$	diazotroph subsistence N quota
$Q^{\mathrm{P}}_{0,\,\mathrm{phy}}$	0.00096 – 0.00296	0.00173	$\mathrm{mol\,(mol\,C)^{-1}}$	non-N_2 fixing phytoplankton subsistence P quota
$Q^{\mathrm{P}}_{0,\,\mathrm{dia}}$	0.002 – 0.00333	0.00236	$\mathrm{mol\,(mol\,C)^{-1}}$	diazotroph subsistence P quota
$k_{\mathrm{Fe,\,phy}}$	0.017 – 0.057	0.03942	$\mu\mathrm{mol\,m^{-3}}$	non-N_2 fixing phytoplankton half-saturation constant for Fe
$k_{\mathrm{Fe,\,dia}}$	0.116 – 0.156	0.13617	$\mu\mathrm{mol\,m^{-3}}$	diazotroph half-saturation constant for Fe
$g_{\max}$	1 – 2	1.62938	$\mathrm{d^{-1}}$	zooplankton maximum specific ingestion rate
ϕ_{phy}	90 – 130	126.91751	$\mathrm{m^3\,(mol\,C)^{-1}}$	capture coefficient of non-N_2 fixing phytoplankton
ϕ_{dia}	113 – 220	130.12582	$\mathrm{m^3\,(mol\,C)^{-1}}$	capture coefficient of diazotrophs
ϕ_{det}	42 – 122	116.62426	$\mathrm{m^3\,(mol\,C)^{-1}}$	capture coefficient of detritus
ϕ_{zoo}	78 – 178	158.25295	$\mathrm{m^3\,(mol\,C)^{-1}}$	capture coefficient of zooplankton
$\lambda_{0,\,\mathrm{phy}}$	0.016 – 0.036	0.01807	$\mathrm{d^{-1}}$	specific mortality rate
wdd	0.052 – 0.066	0.06068	$\mathrm{d^{-1}}$	increase of sinking speed with depth
ν_{det}	0.062 – 0.072	0.07023	$\mathrm{d^{-1}}$	remineralisation rate
